# Linking climate and infectious disease trends in the Northern/Arctic Region

**DOI:** 10.1038/s41598-021-00167-z

**Published:** 2021-10-19

**Authors:** Yan Ma, Georgia Destouni, Zahra Kalantari, Anna Omazic, Birgitta Evengård, Camilla Berggren, Tomas Thierfelder

**Affiliations:** 1 Department of Physical Geography, Stockholm University, 106 91 Stockholm, Sweden; 2 Bolin Centre for Climate Research, Stockholm University, 106 91 Stockholm, Sweden; 3 Department of Chemistry, Environment, and Feed Hygiene, National Veterinary Institute, 751 89 Uppsala, Sweden; 4 Department of Clinical Microbiology, Umeå University, 901 87 Umeå, Sweden; 5 Capio Medical Center Kungsholmen, 112 21 Stockholm, Sweden; 6 Department of Energy & Technology, Swedish University of Agricultural Sciences, 750 07 Uppsala, Sweden

**Keywords:** Climate change, Infectious diseases

## Abstract

Recognition of climate-sensitive infectious diseases is crucial for mitigating health threats from climate change. Recent studies have reasoned about potential climate sensitivity of diseases in the Northern/Arctic Region, where climate change is particularly pronounced. By linking disease and climate data for this region, we here comprehensively quantify empirical climate-disease relationships. Results show significant relationships of borreliosis, leptospirosis, tick-borne encephalitis (TBE), Puumala virus infection, cryptosporidiosis, and Q fever with climate variables related to temperature and freshwater conditions. These data-driven results are consistent with previous reasoning-based propositions of climate-sensitive infections as increasing threats for humans, with notable exceptions for TBE and leptospirosis. For the latter, the data imply decrease with increasing temperature and precipitation experienced in, and projected for, the Northern/Arctic Region. This study provides significant data-based underpinning for simplified empirical assessments of the risks of several infectious diseases under future climate change.

## Introduction

There are indications of climate change driving spatiotemporal shifts in incidence for certain diseases^1–5^. Identification of such climate-sensitive infections (CSIs) is crucial for mitigating climate-driven disease threats. In the Northern/Arctic Region, climate change is particularly rapid and severe^1,2,6^, as ecosystems change^7,8^ and animals move towards the North Pole^3^, bringing microorganisms new for the territories, some of which can cause infections in humans (i.e., zoonoses), causing outbreaks of different magnitude such as epidemics or pandemics. Previous studies have reasoned about potential CSIs, such as borreliosis and tick-borne encephalitis (TBE), based on theoretical, laboratory, and mainly local disease incidence indications^9–11^. However, it is unknown whether these sensitivity estimates are supported by empirical data for climate and disease outbreaks on a large scale, such as over the Northern/Arctic Region. Data-driven identification of emerging disease relationships to observed climate change can be used to test more qualitative, theoretical, and local climate-sensitivity hypotheses and implications towards more accurate, evidence-based, and potentially simplified assessments of disease threats in future scenarios.

In this study, we used synchronous climate and disease incidence data for the Northern/Arctic Region for such data-driven identification of observation-based disease co-variations with recent and ongoing climate change in the region. Disease data were compiled in a regional dataset for seven zoonotic diseases (borreliosis, tularemia, leptospirosis, Q fever, TBE, Puumala virus infection, cryptosporidiosis) caused by pathogenic microorganisms using different vectors in ecosystems to infect humans (Table 1). These data are all derived from diagnosed cases using laboratory confirmation, and cover six Northern/Arctic countries or country parts (Greenland, Iceland, Norway, Sweden, Finland, and parts of northern Russia), distributed across 32–86 regional districts with the longest disease records (from 1969 to 2016)^12^. Along with the disease data, we compiled synchronous data for 22 different climate variables, considering possible climate change impacts on host–pathogen systems^13^ and including several primary (bio)climate variables^14^. These include annual mean, maximum, and minimum monthly and seasonal temperature and precipitation, along with temperature and precipitation in the warmest, coldest, wettest, and driest quarter of each year. Monthly values of these climate variables were obtained from open-access high-resolution gridded datasets of the Climate Research Unit (CRU)^15^, and aggregated to the relevant spatiotemporal scale for linking with corresponding disease data.

**Table 1:** Basic information on the targeted zoonotic infectious diseases^16–18^.

Infection agent	Disease	Transmission pathways
Bacteria	Borreliosis	By vector *Ixodidae* ticks
	Tularemia	Multiple transmission modes: vector-borne (mosquitoes, horseflies, ticks); direct contact; oral; airborne; water-borne
	Leptospirosis	Main hosts are rodent species in natural foci, and livestock and dogs in anthropurgic foci. Leptospira follow the fecal–oral transmission mechanism via water. Humans are usually infected during contact with water contaminated with animal waste
	Q fever	The main reservoirs are farm animals and pets, and transmission to human is mainly through inhalation of contaminated aerosols
Virus	Tick-borne encephalitis (TBE)	By vector *Ixodidae* ticks
	Puumala virus infection	By inhalation of infected rodent excreta
Parasite	Cryptosporidiosis	By ingestion of cryptosporidium oocysts

The statistical relationships between reported disease and climate data were analyzed using Spearman’s correlation coefficient and stepwise regression (significance level *p* < 0.01; see further “Methods”). Consistent spatial aggregation of both climate and disease data over various scales was also used to distinguish a possible emerging large-scale signal from the noise of geographic/spatial variability in the climate-disease relationships. The different scales considered were sub-national, national, and the south/north parts of and the whole multi-national Northern/Arctic Region spanned by the different countries/country parts with data. Such multi-scale exploration of climate-disease relationships reveals their local variability, the degree to which this is dampened on larger scales to reveal a clearer overarching regional relationship pattern, and the representativeness of this pattern for various parts of the region.

## Results and discussion

### Climate change in the Northern/Arctic Region

**Figure 1:**
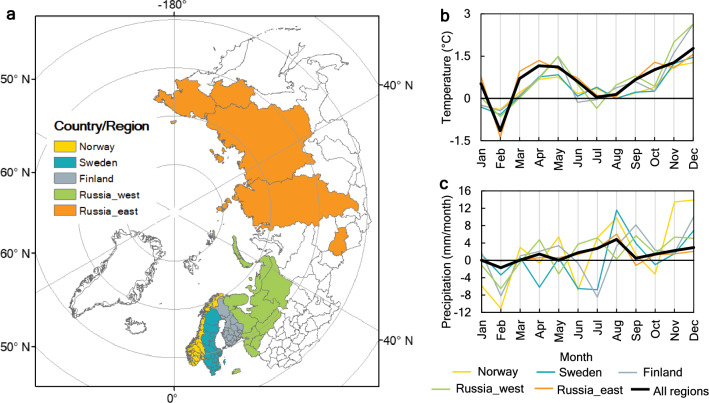
(**a**) The Northern/Arctic Region with countries/country parts represented by the data, together with the associated monthly (**b**) temperature and (**c**) precipitation changes from 1995–2005 to 2005–2015, represented by subtracting the average value of the former period from the average of the latter. The map was generated using ArcGIS 10.5.1 (https://www.esri.com/en-us/home).

During the entire study period, determined by relevant data coverage (1995–2015), the climate has overall become warmer and wetter across the Northern/Arctic Region (Fig. 1a), with mean annual temperature and precipitation both increasing from the first (1995–2005) to the second (2005–2015) half of the period (Supplementary Fig. S1a,b). Changes in average monthly values differ among months and seasons (Fig. 1b,c; Supplementary Fig. S1c,d). All countries or areas studied, i.e., those with relevant data availability in the region (Fig. 1a), display similar patterns of change in monthly temperature, with the highest increases during March-June and September-December, and declines around February (Fig. 1b). This implies warming in spring, summer, and autumn, but little change in average winter temperature (Supplementary Fig. S1c). The seasonal changes in precipitation vary more, including in direction of change, among countries or country parts than temperature does (Fig. 1c; Supplementary Fig. S1d). On average, the precipitation changes are relatively small across the study region, although still with considerable overall increases emerging during June–August and November–December.

### Changes in annual incidence of diseases

**Figure 2:**
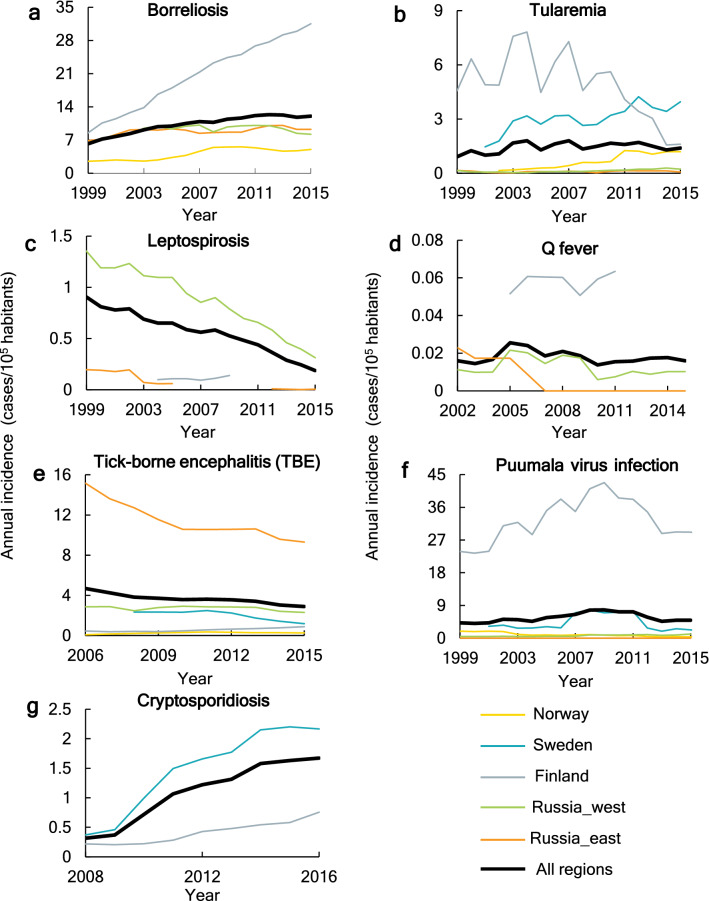
Annual incidence of the seven target infectious diseases over the whole Northern/Arctic Region and in different countries or country parts within the region. The plots show 5-year running average incidence.

Disease incidences are unevenly distributed among the countries/country parts studied across the Northern/Arctic Region. For example, annual incidences of TBE in eastern Russia (Fig. 2e) and that of Puumala virus infection in Finland (Fig. 2f) are markedly higher than elsewhere in the region. Furthermore, average incidence levels vary between the diseases, with Q fever having the lowest annual incidence level (5-year running mean) of less than 0.1 cases per 100,000 inhabitants (Fig. 2d). In general, the incidences of borreliosis (Fig. 2a) and that of cryptosporidiosis (Fig. 2g) show an increasing trend, both nationally and regionally (Supplementary Fig. S2), while that of leptospirosis (Fig. 2c) and TBE (Fig. 2e) show region-scale decline (black lines) but with some regional increases in certain countries (thin colored lines; see also Supplementary Fig. S2). The other diseases show no clear regional trends in incidence, but rather variations around more or less stable region-average levels (Fig. 2b,d,f; Supplementary Fig. S2).

**Figure 3:**
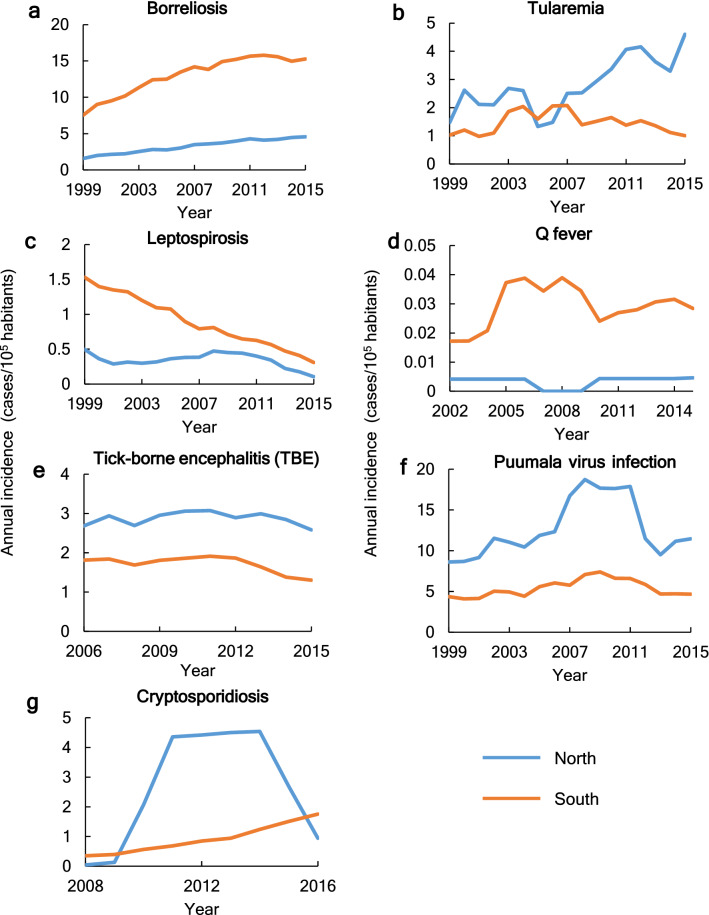
Annual incidence of the seven target infectious diseases in northern and southern parts of the European side of the study region. The parts were divided by the latitude 63°N. The plots show 5-year running average incidence.

Disease incidence level and trends may also differ between the northern and southern parts of the study region, divided by latitude 63°N (Supplementary Fig. S3). The incidences of tularemia, TBE, and Puumala virus infection are higher in the north than in the south (Fig. 3b,e,f), even though the southern part has considerably higher population density (Supplementary Fig. S4). Regarding change trends, borreliosis, leptospirosis, TBE, and Puumala virus infection change in the same direction in both parts as over the whole region (Fig. 3a,c,e,f, Supplementary Fig. S5). This implies that the whole-region trend is representative of a general change pattern for these diseases over the study region. In contrast, the incidence of tularemia changes in opposite directions in the northern and southern parts of the region (Fig. 3b). This implies a more considerable geographic variability for this disease, which is dampened and masked in large-scale averaging so that the whole-region trend becomes small and hardly noticeable. This may also indicate that the spatial foci of this disease have expanded or shifted within the region. Regarding Q fever, this is more common in the south (Fig. 3d), so the overall whole-region trend is dominated by the change trend in the south, while cryptosporidiosis shows several striking temporal peaks in the north, and a relatively low increasing change trend in the south (Fig. 3g), which in combination lead to the clearly increasing whole-region trend for this disease.

### Correlations between the diseases and climate variables

In the whole-region analysis, six of the seven target diseases (borreliosis, leptospirosis, TBE, Puumala virus infection, cryptosporidiosis, Q fever) show significant relationships with multiple climate variables, with Spearman’s correlation coefficient $0.61\le \left|\rho \right|\le 0.98$, *p* < 0.01 (Supplementary Table S1). Specifically, incidence of borreliosis, Puumala virus infection, and cryptosporidiosis show strong positive relationships with autumn temperature (Spearman’s $\rho$ = 0.75), annual maximum monthly precipitation (ρ = 0.82), and mean temperature of the wettest quarter (ρ = 0.85). TBE and leptospirosis, both of which decrease over the study period, show negative relationships with spring precipitation (ρ = − 0.98) and spring temperature (ρ = − 0.95). Q fever shows no obvious trend in incidence, but is still correlated negatively with annual minimum monthly precipitation (ρ = − 0.71). Tularemia does not show significant correlations with any selected climate variable.

These results are to some degree consistent with qualitative assessment propositions for climate sensitivity of various diseases in Europe^9^. However, TBE and leptospirosis exhibit decreases, rather than increases, under the overall warming and wetting trends actually experienced in the Northern/Arctic Region over the study period with data availability, with these findings discussed further below. Tularemia has been identified as a possible CSI in other assessments^9,19^ but does not emerge as such in the present whole-region analysis. This is likely due to the large variability in disease change trends, which are also in opposite directions in different parts of the region and thereby counteract each other in the region-scale averaging of local trends. This counteracting trend variability is, for example, seen between the southern and northern trends in later years in Fig. 3, and has also been reported for tularemia over different parts of Sweden^20^.

**Figure 4:**
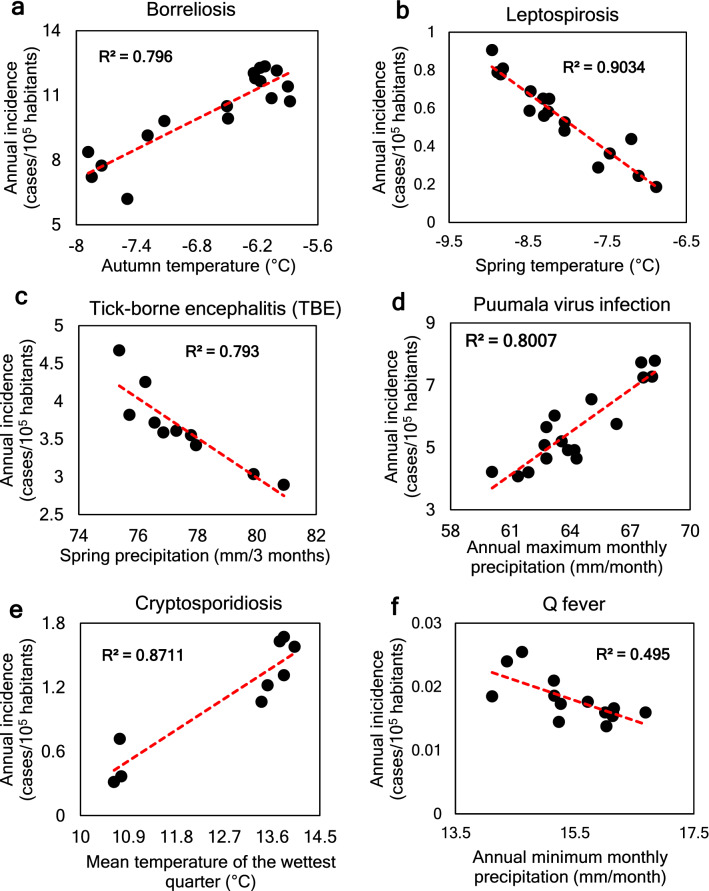
Scatter plots of annual incidence of infectious diseases in the Northern/Arctic Region, as a function of the climate variables with the highest R^2^ value (5-year running means).

Figure 4 shows the climate variables that emerge from the statistical analysis as being most closely related to the incidence of each CSI, with the analysis also including stepwise regression to avoid mutual correlation between variables (Supplementary Table S2). The results indicate the following ranking of the strongest identified proxy climate-disease relationships (R^2^ ≥ 0.8) for five of the seven target CSIs studied: borreliosis with autumn temperature (R^2^ = 0.8, Fig. 4a); leptospirosis with spring temperature (R^2^ = 0.9, Fig. 4b); TBE with spring precipitation (R^2^ = 0.8, Fig. 4c); Puumala virus infection with annual maximum monthly precipitation (R^2^ = 0.8, Fig. 4d); and cryptosporidiosis with mean temperature of the wettest quarter (R^2^ = 0.9, Fig. 4e). Q fever correlates only with annual minimum monthly precipitation and with a weak correlation (R^2^ = 0.5) (Fig. 4f).

The strongest disease correlations with climate variables are thus temperature-related for borreliosis and leptospirosis, water-related for Puumala virus infection, TBE and Q fever, and both temperature and water (hydro-climatically) related for cryptosporidiosis. The negative relationships with hydro-climate observed for leptospirosis (Fig. 4b) and TBE (Fig. 4c) imply decreases, rather than increases, in these diseases with the temperature and precipitation increases experienced over the two decades of the study period (1995–2015) and also projected for the future climate over the study region^2^.

In further analysis of the northern and southern parts of the region, somewhat different climate-disease correlations might be expected due to the various disease change trends exhibited in the different smaller-scale parts (Fig. 3). For example, TBE shows no clear climate sensitivity at the smaller scales due to the relatively small incidence trends at these scales. In combination, however, the similarly directed smaller-scale trends lead to a clear decreasing trend over the whole region. Tularemia, with more highly variable smaller-scale trends, including in opposite directions, emerges as correlated with spring temperature in the north, due to a clear increasing trend in annual incidence in this part of the region (Supplementary Table S2). This is counteracted by the decrease trend exhibited in the southern part of the region and thereby masked in the aggregated whole-region trend. Borreliosis shows fully consistent whole-region and smaller-scale correlations with the same two climate variables, autumn and spring temperature (Supplementary Table S2). This indicates a likely strong sensitivity of borreliosis to these two variables, persisting across different spatial scales and parts of the region.

### Comparison with evidence from previous studies

The results of our large-scale data analysis show some consistency with local observations and mechanical explanations, but also differences due to likely unrelated factors. At whole-region scale, the incidences of TBE are negatively correlated with all (hydro-)climate variables, while those of borreliosis are positively correlated with all climate variables. This is despite the fact that these diseases share the same vector, *Ixodidae* ticks, for which increased annual temperature is reported to increase incidences of tick bites^21^ and the geographic range of ticks, due to the expanded geographic range of associated vegetation communities and mammals caused by a prolonged vegetation period^22^. Some researchers have also argued that the reporting of tick bites has increased due to increased public awareness^23^ and more time spent outdoors^24^, along with the changes in climate, tick bites, and tick range. The decreasing trend in TBE incidence, in spite of these tick increase drivers, might therefore be explained by other, counteracting societal factors, such as vaccination rate co-increasing along with the climate and disease-report factors.

A previous study concluded that leptospirosis is positively correlated with rainfall and temperature^25^. However, in consistency with findings in other previous studies^26^, the results of our data-driven analysis for cryptosporidiosis show significant positive correlations with temperature variables, but negative correlation with annual maximum precipitation. Climate-independent societal factors, such as improved sanitation and increased public awareness, may also play a role for the present empirical findings of decreased leptospirosis under the overall warming and wetting experienced in the study region.

The predominantly water-driven increase in Puumala virus infection with increasing summer and autumn precipitation is consistent with reported positive correlations of the rodent disease-host population with heavy rainfall^27^. The significant infection increase with higher temperature of the driest quarter is likely also hydro-climatically related, consistent with less snow cover during milder winters, when decreasing host protection forces the disease hosts closer to human settlements^28^.

Q fever has been found to be related to droughts^10^ due to its wind-borne transmission pathway, and mainly emerges following droughts. The negative correlation observed here between Q fever and annual minimal precipitation is to some degree explainable by this mechanism.

### Limitations of the study

A primary limitation of this study is that reported laboratory diseases data may not represent actual infection in the community, because some of the infections are mild or even subclinical, so that infected people might not seek healthcare. To estimate the true prevalence of infections, serology would need to be used as a complement in population-based studies. However, serological tests are only performed in specific studies and are not currently used as a tool for monitoring transitions in infectious diseases. In addition, some diseases are not notifiable in all countries because of the significant differences in the registration of epidemic data historically^29^, which requires more efforts to obtain such data for comparable studies across borders.

A second limitation is that the results do not reveal the mechanistic causal relationships that underlie the statistical correlations, and thus need to be interpreted with caution. The strong correlations observed may be caused by either direct or indirect impacts, or even other unrelated factors, and include spurious correlations. However, the focus of this study is on data-based distinction of possible clear long-term, large-scale statistical signals in climate-disease relationships, consistent with the climate-change driver that is, by definition, long-term and large scale, different from the noise of shorter-term, smaller-scale weather-disease variations. This focus implies that the general limitation of statistical correlations versus mechanistic relationships is unavoidable in such a study, and both of these complementary types of analysis are needed and should be compared with each other to move the field forward.

Overall, the large variability in local incidences of the target diseases around and compared with the large-scale region-average conditions (Fig. 2) implies that disease studies performed for different site-specific geographic locations using various scales of supporting data may lead to apparent contradictory or inconsistent results that may or may not be representative of average disease characteristics and change trends emerging over larger regional scales. Across the large regional scale of the entire Northern/Arctic Region (Fig. 1a), our empirical findings suggest significant overarching climate sensitivity of six human diseases (Fig. 4). The negative correlations of TBE and leptospirosis with recent warming and wetting in this region may be surprising and call for further data-driven, large-scale disease studies, as well as targeted mechanistic theoretical and laboratory studies. Climate-independent societal trends, such as general vaccination, sanitation, and public awareness improvements, may coincide with climate change trends and confuse cause-effect attributions for disease trends. This calls for further studies that also include data for such relevant societal factors, as well consistent comparison of disease trends across different countries and regions. However, data-driven studies that link disease and climate trends are useful also in the absence of additional societal data, as they can identify and point out disease trend scenarios without societal mitigation interventions, and thereby support empirically based assessment and prioritization of needs for such interventions.

## Methods

Disease data for the regional dataset are compiled from laboratory reported incidence for the seven zoonotic diseases chosen covering six Northern/Arctic countries and regions (Greenland, Iceland, Norway, Sweden, Finland, and parts of northern Russia). The countries are represented by 32 to 86 districts, with the longest records from 1969 to 2016. We ignored regions with less than 10-year data prior to 2015, and thus retained data only for Norway, Sweden, Finland, and Russia. However, not all diseases are notifiable in all studied countries, for example, borreliosis is not notifiable in Sweden and leptospirosis is not notifiable in Norway^29^. Annual incidence of a disease in each country or in the entire region was calculated as total reported absolute cases divided by total population in selected reporting districts. The time period considered when assessing incidence in the whole region only covered a continuous time sequence ranging from the first year to the latest with at most one country or part of country (eastern part of Russia or western part of Russia). Therefore, Q fever has a time series of annual incidence from 1998 to 2015, TBE from 2002 to 2015, cryptosporidiosis from 2004 to 2016, and the other diseases from 1995 to 2015.

Twenty-two climate variables were selected and calculated from the Climate Research Unit’s (CRU) version 4.04 high-resolution gridded dataset^15^. These were annual mean, maximum, and minimum monthly and seasonal temperature and precipitation, along with temperature and precipitation of the warmest, coldest, wettest, and driest quarter. No missing data appeared in the study region. Grid cells with at least 50% of their area located in the selected districts of the respective disease in the previous step were area-weighted averaged to obtain values for the entire region. The warmest quarter coincides with the summer months (June–August), so in this case the temperature or precipitation of the warmest quarter is represented by the same data as those for the summer. The coldest quarter is also essentially the same as winter (December-February), except in the year 1992/1993, when the coldest quarter was shifted to 1 month earlier than the normal winter period. The wettest quarter varies between summer and autumn months (June–September), and the driest quarter between winter and spring months (January-May).

To make comparisons between the north (low population density) and the south (high population density) for the trends in the diseases and their correlations with climate variables, the European part (excluding eastern Russia) of the study region, based on the centroid of each district, was divided into two parts by latitude 63°N. Annual incidence of diseases and climate data for the two constituent southern and northern parts were calculated with the same methods as for the whole region.

Spearman correlation analysis was conducted to investigate relationships between diseases and climate variables. Statistical significance was set to *p* < 0.01. We applied a 5-year running mean filter to both datasets, on the one hand to be consistent with the “long-term” concept of climate and on the other hand to avoid lack of freedom in the later regression. We then used stepwise (combined forward and backward) regression analysis to identify statistically significant variables contributing to variations in the incidence of each disease, while minimizing the effect of collinearity among variables. Candidate climate variables fed into stepwise regression are those with significance level *p* < 0.01 in Spearman correlation analysis. A variable is considered for addition or subtraction based on the significance level, which was again set at *p* < 0.01.

## Supplementary Information

Supplementary Information.

## Data Availability

All the data used in our analyses are available online. Data on the epidemiology and geography of infectious diseases are published in the CLINF GIS Public Data Repository (https://clinf.org/home/clinf-geographic-information-system/). Monthly data on the climate variables were obtained from high-resolution gridded datasets of the Climate Research Unit (CRU) (https://catalogue.ceda.ac.uk/uuid/89e1e34ec3554dc98594a5732622bce9), and aggregated to the relevant spatiotemporal scale for linking with corresponding disease data.
