# Supplementary material for: Linking climate and infectious disease trends in the Northern/Arctic Region
Source: Sci Rep. 2021 Oct 19;11:20678. doi: 10.1038/s41598-021-00167-z (PMC8526576; doi:10.1038/s41598-021-00167-z)
Supplement: Supplementary file 1 — Supplementary Information. [file 41598_2021_167_MOESM1_ESM.pdf]

Supplementary Materials

Supplementary Figures S1-S5

Supplementary Tables S1-S2

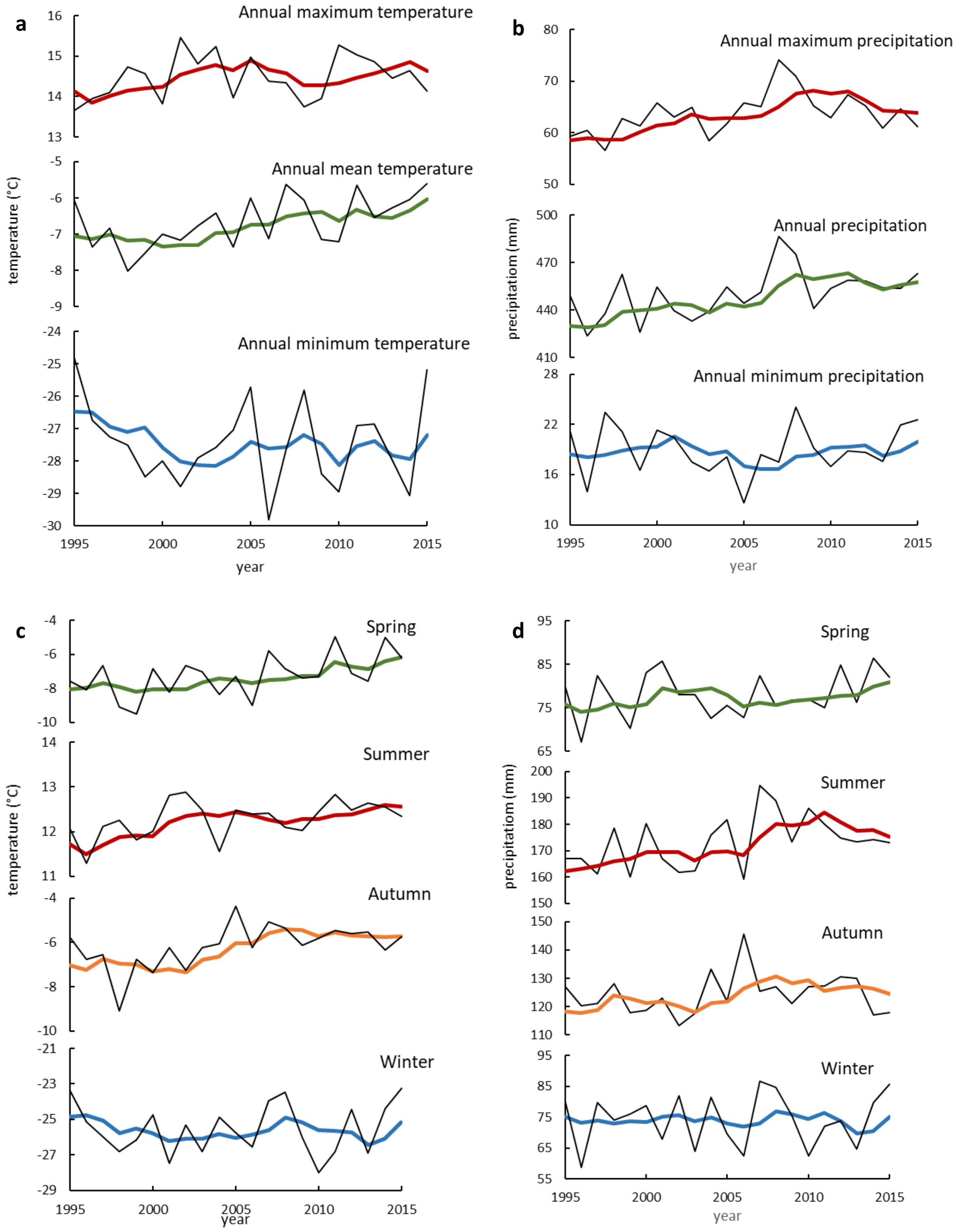

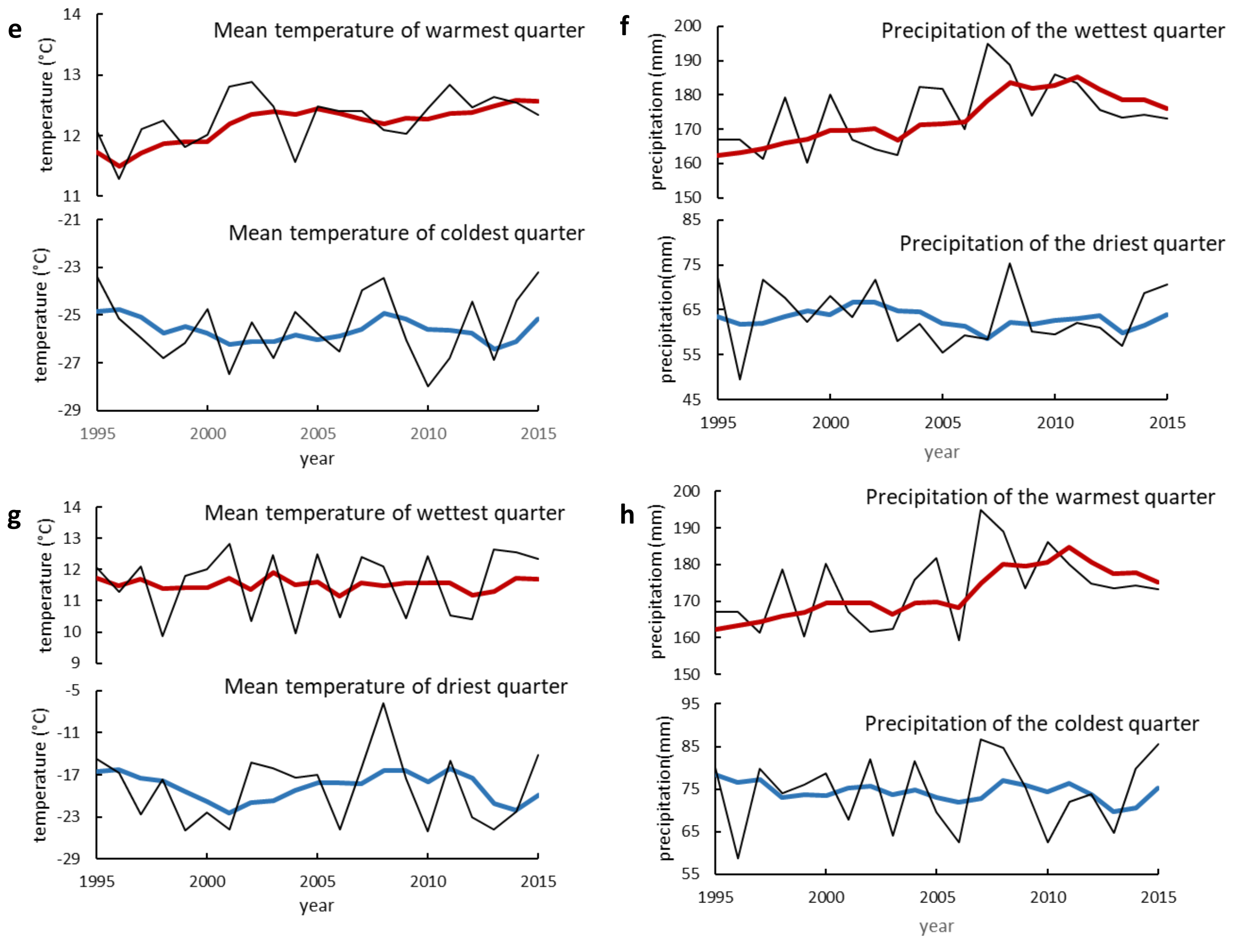

Supplementary Figure S1. Changes in the 22 selected climate variables in the whole Northern/Arctic Region during the period 1995-2015. Black lines are annual values and colored lines are values smoothed with a 5-year running mean filter.

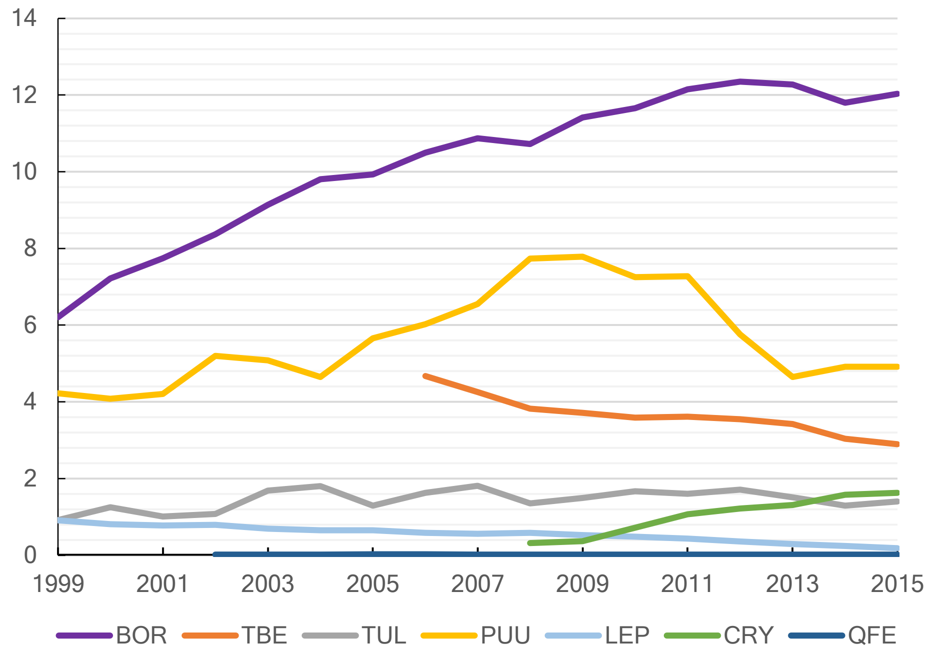

Supplementary Figure S2. Annual incidence of each zoonotic infectious disease in the whole Northern/Arctic Region, 1999-2015. All data smoothed with a 5-year running mean filter. BOR = borreliosis, BRU = brucellosis, CRY = cryptosporidiosis, LEP = leptospirosis, PUU = Puumala virus infection, QFE = Q fever, TBE = tick-borne encephalitis, TUL = tularemia.

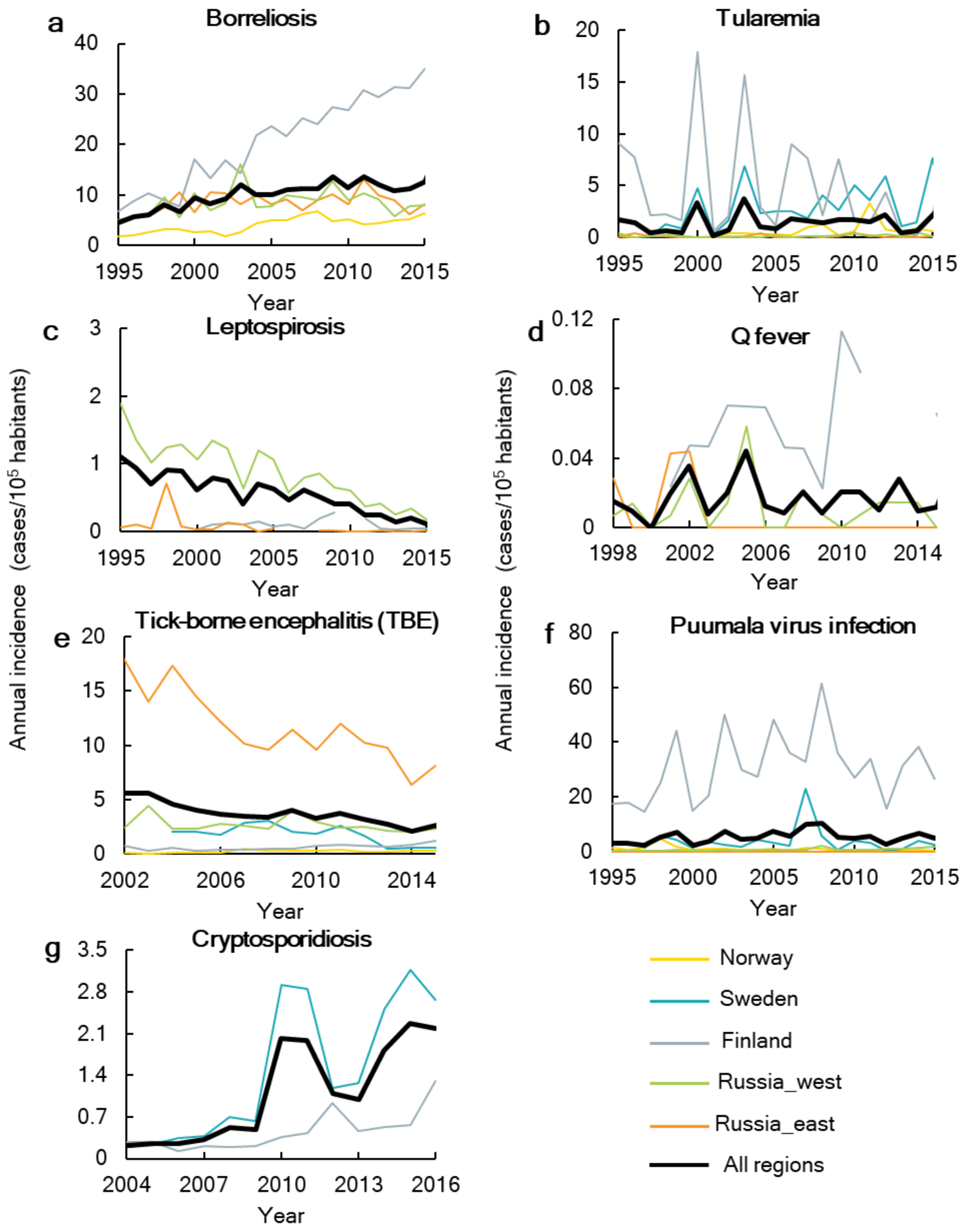

Supplementary Figure S3: Annual incidence of seven infectious diseases over the whole Northern/Arctic Region and different countries or country parts within it.

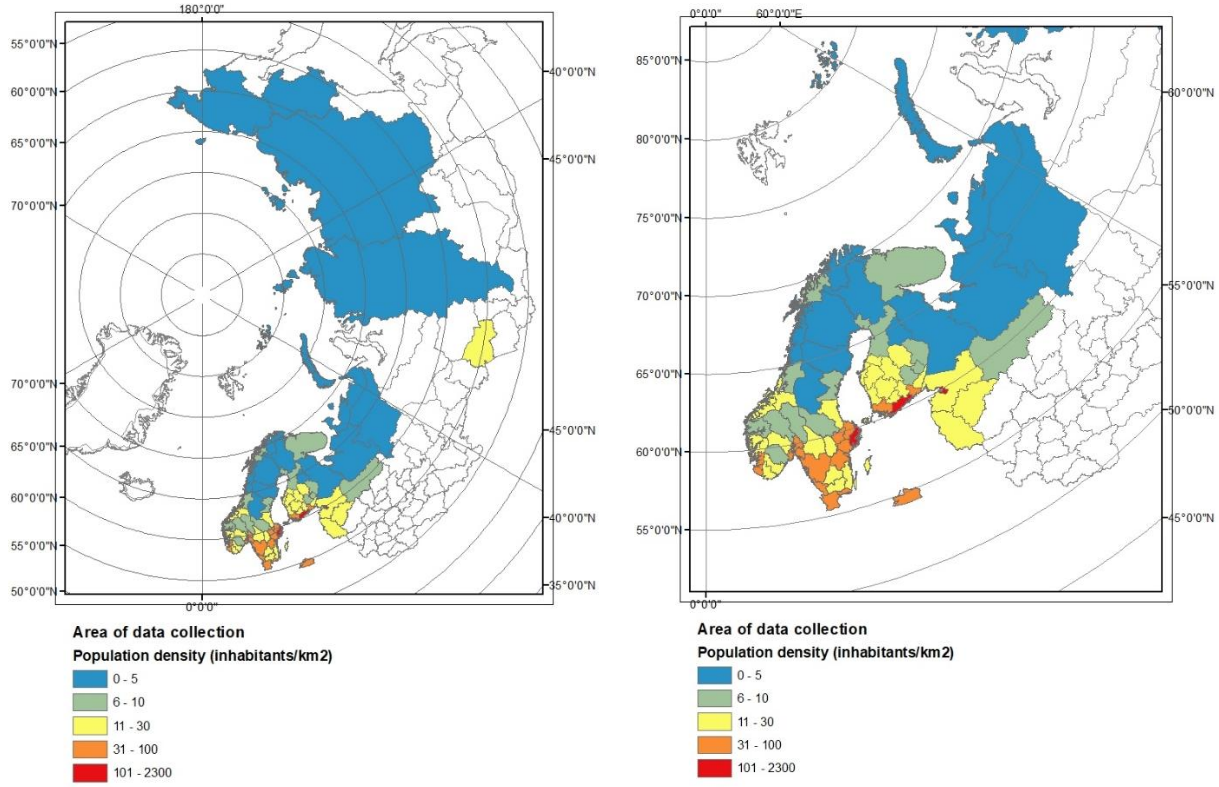

a b

Supplementary Figure S4. Population density based on the average population from 1995 to 2015 in each district of (a) the whole study region, and (b) the European part. The map was generated using ArcGIS 10.5.1 (https://www.esri.com/en-us/home).

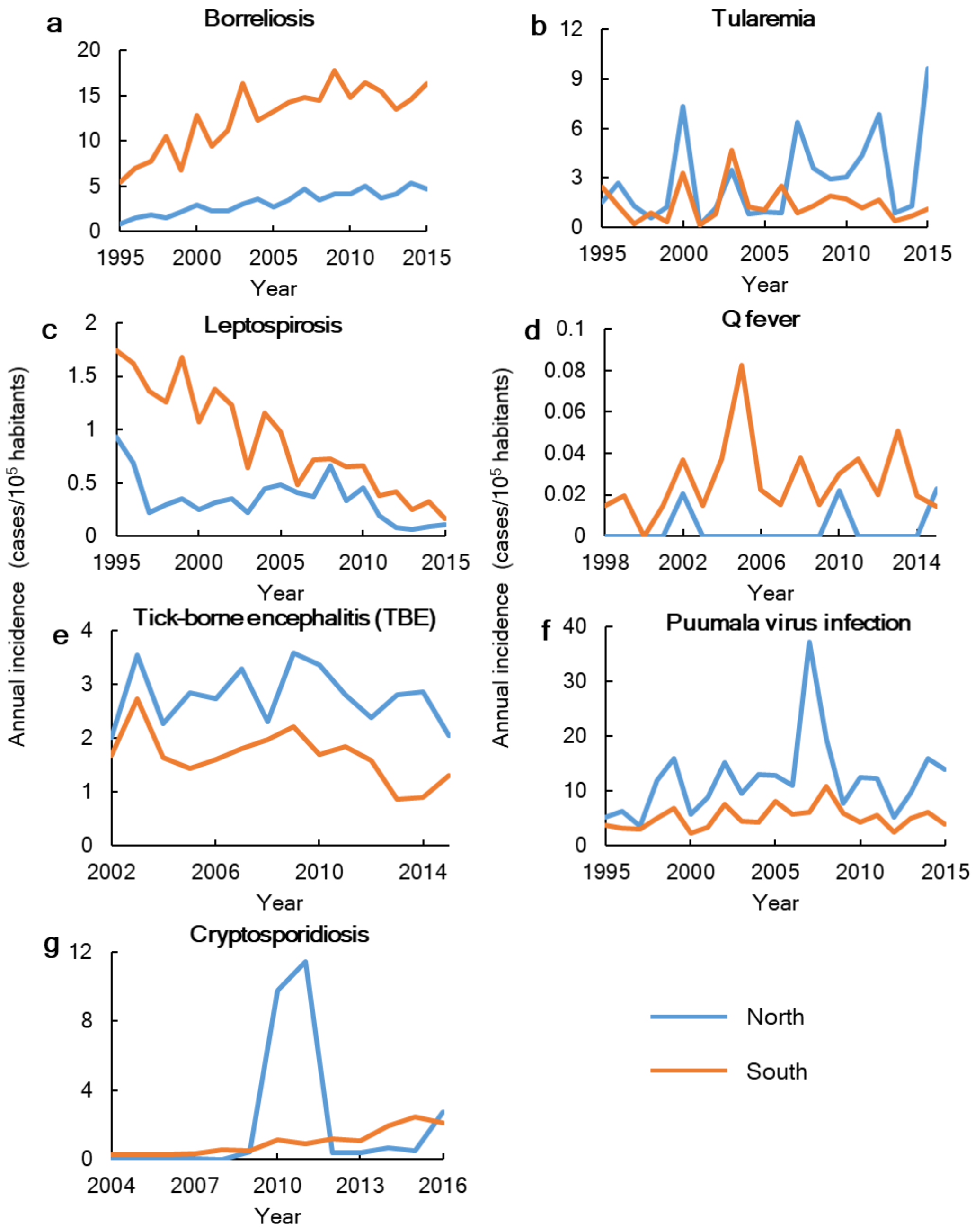

Supplementary Figure S5. Annual incidence of the seven infectious diseases over northern and southern parts in the European side of the study region. The two parts were divided by latitude 63°N.

Supplementary Table S1. Spearman's correlation coefficient *ρ* for all zoonotic infectious diseases and climate variables. BOR = borreliosis, TBE = tick-borne encephalitis, TUL = tularemia, PUU = Puumala virus infection, LEP = leptospirosis, CRY = cryptosporidiosis, QFE = Q fever. *Significant at p<0.01.

|ρ|	>0.8	0.6~0.8	0.4~0.6	0.2~0.4	0~0.2

BOR	Temperature (tmp)										
	Year	Winter	Spring	Summer	Autumn	Yearmax	Yearmin	Warmest quarter	Coldest quarter	Tmp of wettest quarter	Tmp of driest quarter
Norway_Finland_Russia	0.85*	0.17	0.91*	0.56	0.75*	0.17	0.24	0.56	0.17	-0.04	0.23
North	0.81*	0.51	0.9*	0.41	0.84*	-0.08	0.40	0.41	0.41	-0.76*	-0.67*
South	0.60	-0.08	0.8*	0.53	0.78*	0.20	-0.54	0.53	-0.15	-0.17	-0.82*
	Precipitation (pre)										
	Year	Winter	Spring	Summer	Autumn	Yearmax	Yearmin	Wettest quarter	Driest quarter	Pre of warmest quarter	Pre of coldest quarter
Norway_Finland_Russia	0.78*	-0.07	0.22	0.77*	0.61	0.78*	-0.10	0.78*	-0.51	0.77*	-0.18
North	0.64*	-0.38	0.38	0.37	0.55	-0.05	0.14	0.44	-0.50	0.37	-0.77*
South	0.79*	-0.46	0.17	0.79*	0.88*	0.59	-0.44	0.74*	-0.49	0.79*	-0.59

TUL	Temperature (tmp)										
	Year	Winter	Spring	Summer	Autumn	Yearmax	Yearmin	Warmest quarter	Coldest quarter	Tmp of wettest quarter	Tmp of driest quarter
All regions	0.36	0.12	0.44	0.23	0.47	0.18	-0.14	0.23	0.12	-0.10	0.42
North	0.48	0.22	0.78*	-0.12	0.73*	-0.39	0.10	-0.12	0.06	-0.47	-0.72
South	0.33	0.08	0.13	0.45	0.19	0.50	-0.21	0.45	0.00	0.05	-0.05
	Precipitation (pre)										
	Year	Winter	Spring	Summer	Autumn	Yearmax	Yearmin	Wettest quarter	Driest quarter	Pre of Warmest quarter	Pre of coldest quarter
All regions	0.33	-0.18	-0.01	0.21	0.30	0.46	-0.30	0.35	-0.38	0.21	-0.18
North	0.36	-0.51	0.54	0.35	0.26	-0.54	0.56	-0.19	-0.01	0.35	-0.64
South	-0.23	-0.23	-0.18	0.04	-0.08	0.18	-0.49	-0.05	-0.51	0.04	-0.27

LEP	Temperature (tmp)

	Year	Winter	Spring	Summer	Autumn	Yearmax	Yearmin	Warmest quarter	Coldest quarter	Tmp of wettest quarter	Tmp of driest quarter
Finland_Russia	-0.88*	-0.23	-0.95*	-0.59	-0.7*	-0.15	-0.29	-0.59	-0.23	-0.78*	-0.02
North	0.13	0.47	-0.30	-0.34	0.04	-0.15	0.42	-0.34	0.49	0.13	-0.02
South	-0.78*	-0.04	-0.81*	-0.47	-0.81*	-0.11	0.48	-0.47	0.06	-0.63*	0.44
	Precipitation (pre)										
	Year	Winter	Spring	Summer	Autumn	Yearmax	Yearmin	Wettest quarter	Driest quarter	Pre of Warmest quarter	Pre of coldest quarter
Finland_Russia	-0.75*	-0.18	-0.39	-0.72*	-0.63*	-0.71*	-0.01	-0.71*	0.24	-0.72*	-0.02
North	0.40	0.42	0.19	0.01	0.36	0.77*	-0.28	0.47	0.01	0.01	-0.01
South	-0.79*	0.50	-0.27	-0.57	-0.84*	-0.34	0.04	-0.13	0.53	-0.57	0.58

QFE	Temperature (tmp)										
	Year	Winter	Spring	Summer	Autumn	Yearmax	Yearmin	Warmest quarter	Coldest quarter	Tmp of wettest quarter	Tmp of driest quarter
Finland_Russia	0.01	0.05	-0.30	-0.16	0.13	0.07	0.24	-0.16	0.05	-0.41	0.54
North	0.03	-0.18	0.78*	-0.15	0.24	-0.48	-0.42	-0.15	-0.32	-0.20	-0.32
South	0.40	0.02	-0.37	-0.27	0.55	-0.42	0.18	-0.27	-0.09	0.23	-0.27
	Precipitation (pre)										
	Year	Winter	Spring	Summer	Autumn	Yearmax	Yearmin	Wettest quarter	Driest quarter	Pre of Warmest quarter	Pre of coldest quarter
Finland_Russia	-0.14	-0.45	-0.27	-0.06	0.22	-0.02	-0.71*	-0.05	-0.24	-0.06	-0.45
North	0.15	-0.7*	-0.61	0.71*	-0.04	-0.52	0.56	0.22	-0.03	0.71*	-0.50
South	0.12	-0.45	0.64	0.14	0.09	0.13	-0.78*	0.27	-0.61	0.14	-0.66

TBE	Temperature (tmp)										
	Year	Winter	Spring	Summer	Autumn	Yearmax	Yearmin	Warmest quarter	Coldest quarter	Tmp of wettest quarter	Tmp of driest quarter
All regions	-0.43	0.30	-0.89*	-0.81*	0.33	-0.39	-0.01	-0.81*	0.30	-0.52	0.49
North	-0.48	-0.09	-0.02	-0.42	-0.02	-0.14	-0.25	-0.42	-0.27	0.44	-0.38
South	-0.30	0.01	0.09	-0.18	-0.56	0.02	-0.09	-0.18	0.05	0.28	0.14
	Precipitation (pre)										

	Year	Winter	Spring	Summer	Autumn	Yearmax	Yearmin	Wettest quarter	Driest quarter	Pre of Warmest quarter	Pre of coldest quarter
All regions	-0.05	0.21	-0.98*	-0.19	0.39	0.20	-0.76	0.04	-0.45	-0.19	0.21
North	0.26	-0.05	0.15	0.54	-0.02	-0.01	0.01	0.32	0.07	0.54	-0.41
South	0.26	0.05	-0.19	0.54	-0.14	0.73	-0.19	0.68	0.12	0.54	0.20

PUU	Temperature (tmp)										
	Year	Winter	Spring	Summer	Autumn	Yearmax	Yearmin	Warmest quarter	Coldest quarter	Tmp of wettest quarter	Tmp of driest quarter
All regions	0.60	0.53	0.35	0.04	0.76*	-0.12	0.19	0.04	0.53	-0.04	0.82*
North	0.69*	0.75*	0.23	0.29	0.46	0.24	0.67*	0.29	0.74*	-0.35	-0.04
South	0.63*	0.36	0.30	0.27	0.56	0.07	-0.08	0.27	0.32	0.14	-0.31
	Precipitation (pre)										
	Year	Winter	Spring	Summer	Autumn	Yearmax	Yearmin	Wettest quarter	Driest quarter	Pre of Warmest quarter	Pre of coldest quarter
All regions	0.7*	0.35	-0.35	0.61*	0.63*	0.82*	-0.37	0.75*	-0.49	0.61*	0.35
North	0.72*	0.40	-0.02	-0.30	0.67*	0.40	-0.37	0.74*	-0.64*	-0.30	-0.26
South	0.42	0.00	0.03	0.50	0.29	0.58	-0.46	0.50	-0.50	0.50	-0.18

CRY	Temperature (tmp)										
	Year	Winter	Spring	Summer	Autumn	Yearmax	Yearmin	Warmest quarter	Coldest quarter	Tmp of wettest quarter	Tmp of driest quarter
Sweden_Finland	0.18	-0.45	0.85*	0.10	0.28	0.70	-0.48	0.10	-0.32	0.85*	0.20
North	-0.60	-0.95*	0.35	0.22	-0.13	0.50	-0.82*	0.22	-0.9*	0.62	-0.9*
South	-0.13	-0.45	0.65	-0.35	0.13	0.05	-0.38	-0.35	-0.32	0.85*	0.13
	Precipitation (pre)										
	Year	Winter	Spring	Summer	Autumn	Yearmax	Yearmin	Wettest quarter	Driest quarter	Pre of Warmest quarter	Pre of coldest quarter
Sweden_Finland	-0.38	-0.07	0.30	-0.27	-0.70	-0.97*	0.63	-0.73	0.78	-0.27	0.45
North	-0.15	-0.97*	0.75	0.45	-0.38	-0.10	0.43	-0.37	0.52	0.45	-0.42
South	0.00	0.32	0.15	-0.27	-0.50	-0.93*	-0.13	-0.72	0.63	-0.27	0.65

Supplementary Table S2. Results of stepwise regression of significant variables

Diseases	Regions	Significant variables *	Added/removed	R^2	Adjusted-R^2
Borreliosis	Norway+Finland+Russia	Autumn temperature	addition	0.796	0.782
		Spring temperature	addition	0.896	0.881
	North	Autumn temperature	addition	0.847	0.836
		Spring temperature	addition	0.938	0.930
	South	Autumn temperature	addition	0.798	0.785
		Spring temperature	addition	0.923	0.912
Tick-borne encephalitis	The whole region	Spring precipitation	addition	0.793	0.767
	North	N/A			
	South	N/A			
Tularemia	The whole region	N/A			
	North	Spring temperature	addition	0.621	0.596
	South	N/A			
Q fever	Finland+Russia	Annual minimum monthly precipitation**		0.495	
	North	Winter precipitation	addition	0.193	0.126
	South	Annual minimum monthly precipitation**		0.480	
Puumala virus infection	The whole region	Annual maximum monthly precipitation	addition	0.801	0.787
		Mean temperature of the driest quarter	addition	0.896	0.881
		Summer precipitation	addition	0.941	0.928
	North	Annual precipitation	addition	0.662	0.640
		Precipitation of the driest quarter	addition	0.808	0.781
	South	Annual mean temperature**		0.402	
Leptospirosis	Finland+Russia	Spring temperature	addition	0.903	0.897
	North	Annual maximum monthly precipitation**		0.413	
	South	Autumn temperature	addition	0.814	0.801
		Spring temperature	addition	0.913	0.901

Cryptosporidiosis	Sweden+Finland	Mean temperature of the wettest quarter	addition	0.871	0.853
	North	Mean temperature of the coldest quarter	addition	0.913	0.900
	South	Annual maximum monthly precipitation	addition	0.875	0.857

*Significance for all variables $p < 0.01$.
**The only correlated variable with the significance $p < 0.01$ in Spearman correlation analysis.
